# Predictive value of P wave terminal force in lead V1 for atrial fibrillation: A meta‐analysis

**DOI:** 10.1111/anec.12739

**Published:** 2020-02-05

**Authors:** Zhuoshan Huang, Zhenda Zheng, Bingyuan Wu, Leile Tang, Xujing Xie, Ruimin Dong, Yanting Luo, Suhua Li, Jieming Zhu, Jinlai Liu

**Affiliations:** ^1^ Department of Cardiovascular Medicine The Third Affiliated Hospital Sun Yat‐sen University Guangzhou China

**Keywords:** atrial fibrillation, electrocardiogram, P wave terminal force in lead V1, predictor

## Abstract

**Background:**

Several studies have explored the association between P wave terminal force in lead V1 (PTFV1) and risk of atrial fibrillation (AF) occurrence, but the results were controversial. This meta‐analysis aimed to examine whether abnormal PTFV1 could predict AF occurrence.

**Methods:**

We searched PubMed, Embase, and Cochrane Library databases for articles published before August 25, 2018. Pooled odds ratios (ORs) of AF occurrence were calculated using random‐effects models to explore the significance of PTFV1.

**Results:**

A total of 12 studies examining 51,372 participants were included, with 9 studies analyzing PTFV1 as a categorical variable and 4 studies analyzing PTFV1 as a continuous variable. As a categorical variable, abnormal PTFV1 (>0.04 mm s) was significantly associated with AF occurrence with a pooled OR of 1.39 (95% confidence interval [CI] 1.08–1.79, *p* = .01). Subgroup analysis found that ORs of studies in hemodialysis patients (OR = 4.89, 95% CI 2.54–9.90, *p* < .001) and acute ischemic stroke patients (OR = 1.60, 95% CI 1.14–2.25, *p* = .007) were higher than general population (OR = 1.15, 95% CI 1.03–1.29, *p* = .01). Studies from Europe (OR = 1.05, 95% CI 0.91–1.20, *p* = .51) yielded lower OR of endpoints compared with Asia (OR = 1.89, 95% CI 1.38–2.60, *p* < .001) and United States (OR = 1.43, 95% CI 1.19–1.72, *p* < .001). As a continuous variable, PTFV1 was also significantly associated with AF occurrence with a polled OR per 1 standard deviation (*SD*) change of 1.27 (95% CI 1.02–1.59, *p* = .03).

**Conclusions:**

PTFV1 was significantly associated with the risk of AF and was considered to be a good predictor of AF occurrence in population with or without cardiovascular diseases.

## 1 INTRODUCTION

P wave terminal force in lead V1 (PTFV1) was first mentioned by Morris et al in 1964 (Morris, Estes, Whalen, Thompson, & Mcintosh, 1964). It is calculated by multiplying the P prime duration by the P prime amplitude in lead V1 of electrocardiogram (ECG). Commonly, abnormal PTFV1 (generally defined as PTFV1 > 0.04 mm s) was considered as a reflection of left atrial enlargement. However, several studies demonstrated that PTFV1 was a sign of delayed interatrial conduction, left ventricular fibrosis or left atrial function, indicating that PTFV1 is composed of various factors apart from left atrial size (Josephson, Kastor, & Morganroth, 1977; Tiffany Win et al., 2015). Atrial fibrillation (AF), one of the most common cardiac arrhythmias in adult medicine, is closely associated with stroke (Healey et al., 2012), heart failure (Ling, Kistler, Kalman, Schilling, & Hunter, 2016), cardiovascular mortality, and sudden cardiac death (Benjamin et al., 1998). However, asymptomatic AF is likely underdiagnosed (Lee & Mittal, 2018). Therefore, it is necessary for identifying individuals at high risk of AF from the patients and general populations. Association between PTFV1 and AF has been explored for decades, and several studies have been carried out (Baturova et al., 2016; Eranti et al., 2014; Francia et al., 2015; Goda et al., 2017; Kamel et al., 2014; Lehtonen et al., 2017; Magnani et al., 2015; Nishi, Fujimoto, Hisanaga, Ogawa, & Kitamura, 2013; Rasmussen, Kumarathurai, Fabricius‐Bjerre, Davidsen, & Sajadieh, 2017; Soliman, Prineas, Case, Zhang, & Goff, 2009; Sugiyama, Ohara, Watanabe, Junya, & Daisuke, 2017). However, the results of previous studies were controversial. This meta‐analysis aimed to summarize the relevant literatures to clarify the predictive value of abnormal PTFV1 for risk of AF occurrence.

## 2 METHODS

### 2.1 Search strategy

Our study was performed according to the Preferred Reporting Items for Systematic reviews and Meta‐Analyses for Protocols 2015 (PRISMA‐P 2015) statement (Moher et al., 2015). A systemic electronic search was conducted by cross‐searching PubMed, Embase, and Cochrane Library up to 25 August 2018. The following key words were included: “p wave indices,” “p wave index,” “p wave terminal force,” “p terminal force,” and “atrial fibrillation.” A hand‐searching of reference lists was also performed for searching additional relevant literatures. All published studies in English were considered.

### 2.2 Study selection

Studies were included in the analyses if they met the following criteria: (a) The study design was a prospective cohort, retrospective cohort, or case–control study in human; (b) the exposure of interest contained PTFV1 and the outcomes contained AF occurrence; (c) effect estimates included relative risk (RR), odds ratio (OR) or hazard ratio (HR) with 95% confidence interval (CI), or sufficient data were provided to calculate them.

Studies were excluded on the basis of the following criteria: (a) Study population was all heart disease patients; (b) cutoff value of PTFV1 was not appropriate (eg: PTFV1 ≥ 0.12 mm s) (Ishida et al., 2010); (c) review articles. If study cohorts were found duplicated or articles were duplicate publications (Alonso, Soliman, & Agarwal, 2010; Magnani et al., 2013, 2015; Soliman et al., 2009), the studies with the most detailed data were included in our meta‐analysis.

### 2.3 Data extraction and quality assessment

Two investigators (Dr. Huang and Dr. Zheng) independently extracted information about first author, publication year, geographical location, study population, sample size, mean age, gender, AF patients, diagnostic methods of AF, follow‐up time, and study design for each including study. Any disagreement was discussed until agreement was reached. The Newcastle‐Ottawa Scale (NOS) was used for quality assessment of the included study (Stang, 2010). Two investigators (Dr. Huang and Dr. Zheng) independently performed quality assessment for each study based on the assessment of selection, comparability, and outcome (cohort studies) or exposure (case–control studies), respectively, which allowed a total of 9 points summarizing eight aspects. The higher the score, the higher the quality.

### 2.4 Statistical analysis

Data analysis was performed using Comprehensive Meta‐Analysis software (Version 2). The generic inverse variance method was used to calculate the pooled estimates. The multivariate‐adjusted HR and RR values in multivariate Cox proportional hazards model were directly regarded as OR values to pool together according to the previous literature (Greenland, 1987). As 2 forms of PTFV1 (effect estimates for a 1‐*SD* change as a continuous variable and effect estimates as a categorical variable) were provided in the relevant studies, separate meta‐analyses were conducted to evaluate their association with AF occurrence. Pooled ORs and their 95% CIs were calculated using a random‐effects model in consideration of studies with potentially clinical and methodological diversity, such as differences in study population, diagnostic methods, and follow‐up time. Meanwhile, heterogeneity between studies was quantified using Cochran's Q statistic and the *I* ^2^ statistic. *I* ^2^ value between 0% and 25% indicates insignificant heterogeneity, 26% and 50% indicates low heterogeneity, 51% and 75% indicates moderate heterogeneity, and 76% and 100% indicates high heterogeneity (Higgins, Thompson, Deeks, & Douglas, 2003). Publication bias was assessed by funnel plots, Begg's adjusted rank correlation test, and Egger's regression asymmetry test. Sensitivity analyses by removing one study at a time were performed to identify the source of heterogeneous. Subgroup analysis and meta‐regression analysis was performed separately to investigate if any categorical and continuous variable was associated with study outcomes. Statistical significance was defined as a 2‐tailed *p* value of .05.

## 3 RESULTS

### 3.1 Search results

**Figure 1:**
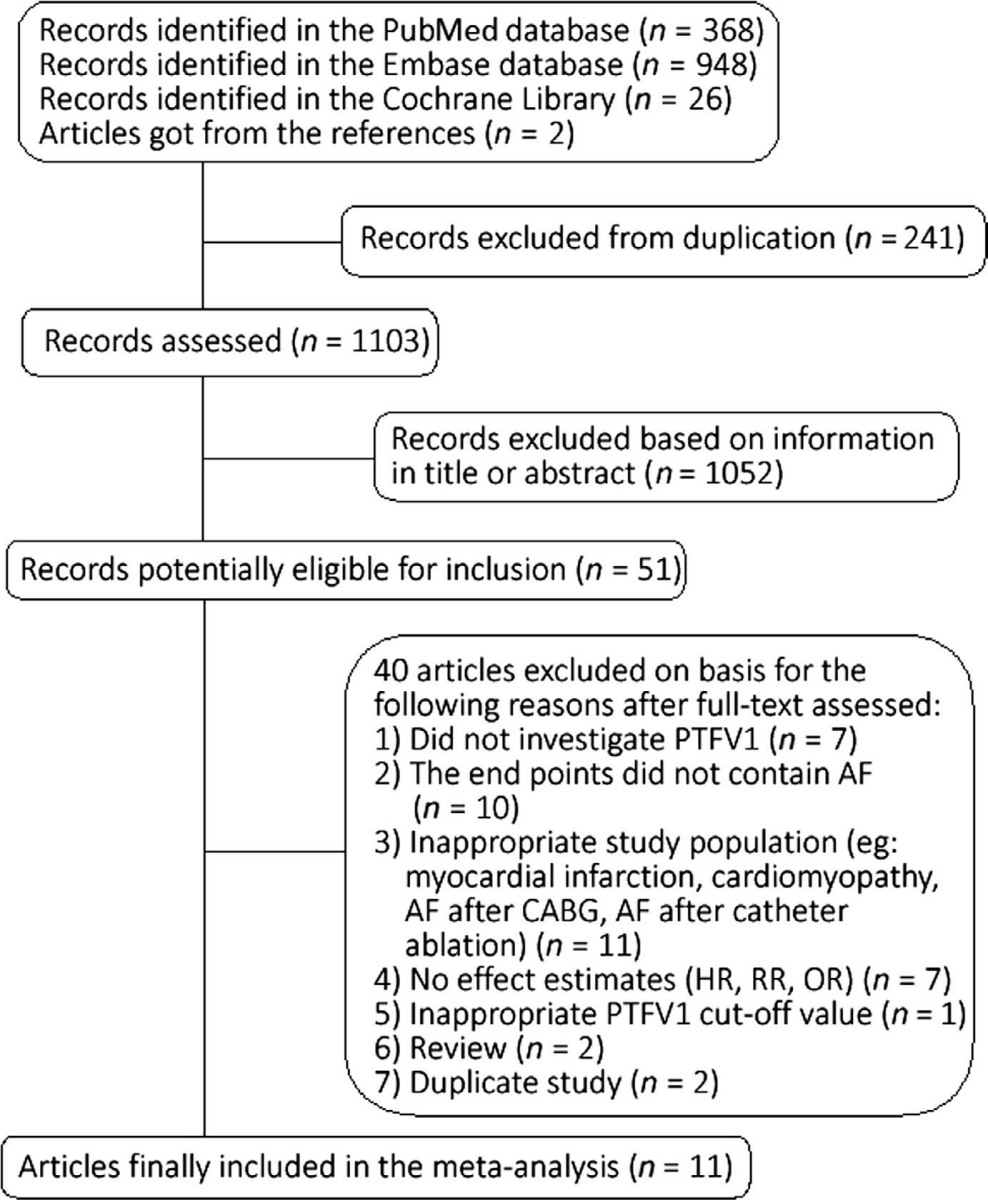
Flowchart of identifying the studies in the meta‐analysis

The study selection process and results from the literature search were shown in Figure 1. A total of 1,344 records (368 from PubMed, 948 from Embase, 26 from Cochrane Library, and 2 from additional reference searching) were identified. After exclusion on basis of title, abstract and/or text, 11 articles containing 12 independent studies (FHS and ARIC studies were reported, respectively, in the same article in 2015 by Dr. Magnani (Magnani et al., 2015)) were finally included in the present meta‐analysis. Among the including studies, 9 studies analyzed PTFV1 as a category variable and 4 studies analyzed PTFV1 as a continuous variable.

### 3.2 Characteristics of the included studies

Our meta‐analysis involved a total of 51,372 participants with a mean age ranging from 44 to 74 years in 12 studies. The proportion of males ranged from 42.7% to 64.2%. Finally, 3,610 cases were diagnosed atrial fibrillation and incidence of AF ranged from 0.7% to 33.3%. Baseline characteristics of these studies were summarized in Table 1. Of studies analyzing PTFV1 as a category variable, abnormal PTFV1 was defined as >0.04 mm s. A total of 44,317 participants with a mean age ranging from 44 to 73 were included. Of studies analyzing PTFV1 as a continuous variable, a total of 22,484 participants with a mean age ranging from 54 to 74 were included. The study quality scores assessed by Newcastle‐Ottawa Scale ranged from 6 to 9.

**Table 1:** Basic characteristic of the included studies

First author/Year	Location	Study population	Sample size (*n*)	Age (years)	No. of Male	No. of AF	AF diagnosis	Follow‐up time (years)	Study design	Quality score
Lehtonen et al. (2017)	Finland	Finnish adult population ≥ 30 years	5,667	51.5 ± 14.1	2,557	423	ICD−10 code I48 in the National Hospital Discharge or Causes of Death registers during follow‐up or AF in the Health 2011 Survey follow‐up ECG	11.9 ± 2.9	Prospective cohort study	8
Goda et al. (2017)	Japan	Acute ischemic stroke patients	226	74.2 ± 11.7	134	16	Bedside ECG monitoring	‐	Cross‐sectional study	7
Sugiyama et al. (2017)	Japan	Acute ischemic stroke patients	105	72.8 ± 13.4	NA	11	24‐hr ECG monitoring	‐	Cross‐sectional study	6
Rasmussen et al. (2017)	Denmark	no apparent heart disease or AF with age between 55–75	678	55–75	NA	77	NA	14	Prospective cohort study	7
Baturova et al. (2016)	United States	Ischemic stroke patients	165	67.3 ± 10.0	106	55	NA	‐	Retrospective study	7
Magnani et al. (2015) (FHS)	United States	General population	3,110	62.6 ± 9.8	1,339	217	ECG, Holter, and tracings at or external to FHS after adjudication by 2 physicians	10	Prospective cohort study	8
Magnani et al. (2015) (ARIC)	United States	General population	8,254	62.3 ± 5.6	3,521	458	Review of hospital discharge records for ICD−9 codes 427.31 or 427.32	10	Prospective cohort study	8
Francia et al. (2015)	Italy	Hypertensive patients	88	67.2 ± 8.0	54	44	Standard or Holter ECG	‐	Case–control study	8
Kamel et al. (2014)	United States	men and women aged 45–84 years who were free of clinically apparent cerebrovascular or cardiovascular disease, including AF	6,741	62.1 ± 10.2	3,174	541	ECG	8.5 (7.7–8.6)	Prospective cohort study	7
Eranti et al. (2014)	Finland	males and females aged 30–59 years who underwent clinical baseline examinations	10,647	43.9 ± 1.3	5,619	1606	Obtained from the Finnish Hospital Discharge Register	35–41	Prospective cohort study	7
Nishi et al. (2013)	Japan	hemodialysis patients	262	62.2 ± 14.2	149	45	ECG	5	Prospective cohort study	7
Soliman et al. (2009)	United States	General population	15,429	52.4 ± 5.8	6,887	117	ECG	6.97 ± 1.46	Prospective cohort study	9

Abbreviations: AF, atrial fibrillation; ARIC, The Atherosclerosis Risk in Communities Study; ECG, electrocardiogram; FHS, Framingham Heart Study.

### 3.3 Relationship between PTFV1 and AF occurrence

**Figure 2:**
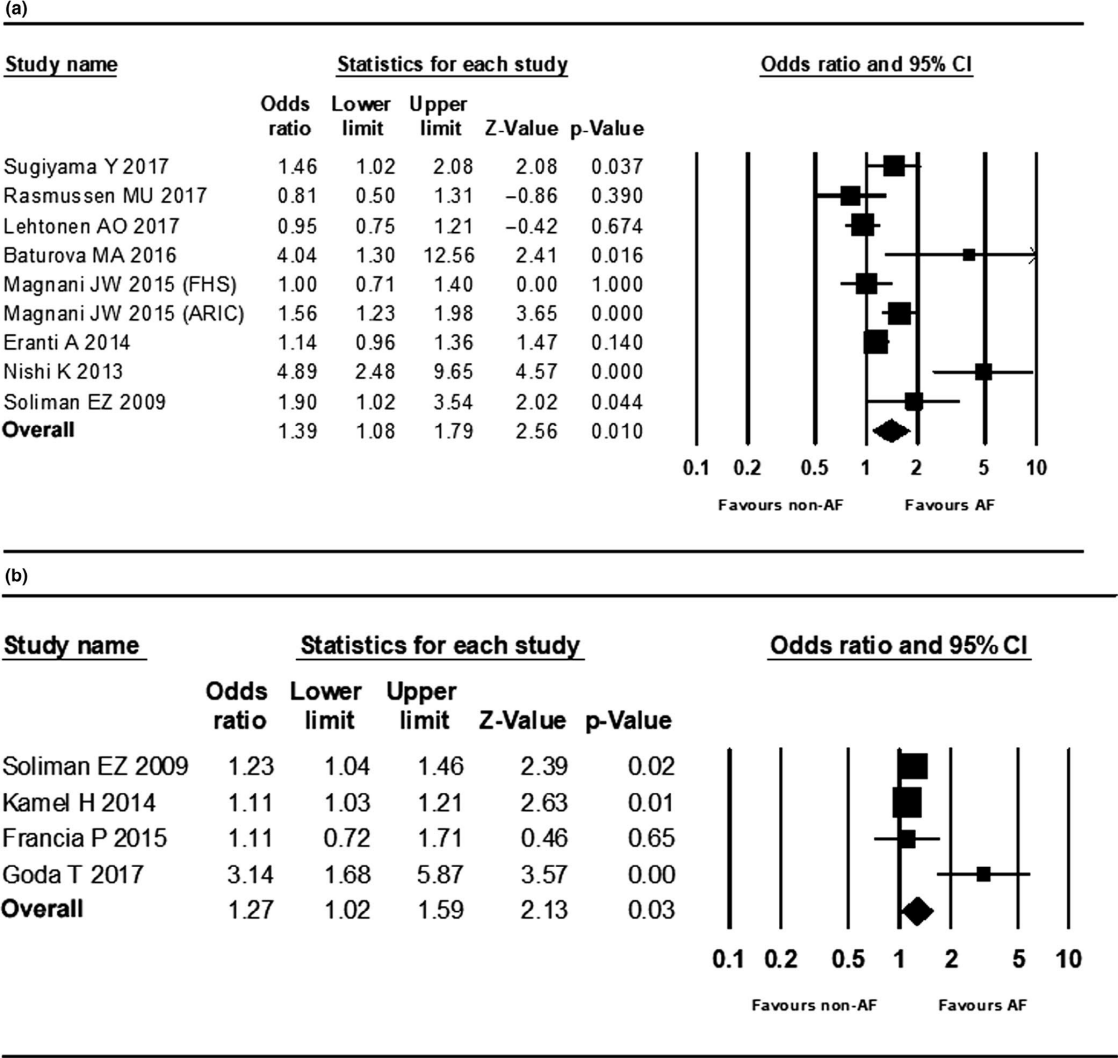
Forrest plots. (a) Forrest plot of studies PTFV1 analyzed as a categorical variable in meta‐analysis. (b) Forrest plot of studies PTFV1 analyzed as a continuous variable in meta‐analysis

**Figure 3:**
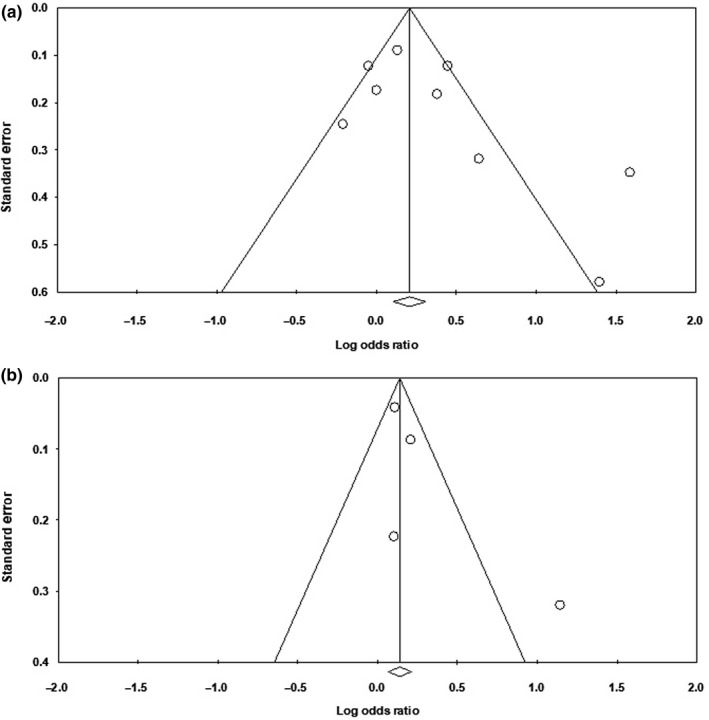
Funnel plots. (a) Funnel plot of studies PTFV1 analyzed as a categorical variable. (b) Funnel plot of studies PTFV1 analyzed as a continuous variable

When PTFV1 was analyzed as a categorical variable, meta‐analysis of 9 included studies (Baturova et al., 2016; Eranti et al., 2014; Lehtonen et al., 2017; Magnani et al., 2015; Nishi et al., 2013; Rasmussen et al., 2017; Soliman et al., 2009; Sugiyama et al., 2017) showed abnormal PTFV1 was significantly associated with AF occurrence with a pooled OR of 1.39 (95% CI 1.08–1.79, *p* = .01) (Figure 2a). The funnel plot of the included studies resembled an inverted funnel, indicating that publication bias was unlikely (Figure 3a). Meanwhile, the Begg's rank correlation test gave a Tau value as 0.33 (*p* = .21) and the Egger's regression intercept is 2.36 (*p* = .13), which suggested that the meta‐analysis was free from publication bias. *I* ^2^ was 77.87% (Q = 36.15, *p* < .001), meaning heterogeneity of studies was relatively high. Furthermore, we conducted sensitivity analyses by removing one study at a time. After excluding Nishi's study (Nishi et al., 2013) which explored PTFV1 and new‐onset AF occurrence in hemodialysis patients, heterogeneity of including studies decreased (*I* ^2^ = 64.89%, Q = 19.94, *p* = .006), while pooled OR reduced to 1.23 (95% CI 1.00–1.50, *p* = .045). To other studies, we did not observe significant change in heterogeneity or pooled OR with these sensitivity analyses.

When PTFV1 was analyzed as a continuous variable, meta‐analysis of 4 included studies (Francia et al., 2015; Goda et al., 2017; Kamel et al., 2014; Soliman et al., 2009) also showed elevated PTFV1 was significantly associated with AF occurrence with a pooled OR per 1‐*SD* change of 1.27 (95% CI 1.02–1.59, *p* = .03) (Figure 2b). The funnel plot of the included studies resembled an inverted funnel (Figure 3b). The Begg's rank correlation test gave a Tau value as 0.67 (*p* = .17) and the Egger's regression intercept is 2.02 (*p* = .26), which suggested that the meta‐analysis was free from publication bias. *I* ^2^ was 72.91% (Q = 11.07, *p* = .011), meaning that the study results were moderated heterogeneous. Since the number of studies included was small, we did not conduct sensitivity and meta‐regression analyses further.

### 3.4 Subgroup analysis and meta‐regression analysis

Subgroup analysis and meta‐regression analysis were performed in studies PTFV1 analyzed as categorical variable. We performed subgroup analysis based on different areas, population, PTFV1 measurement methods, or study types (Table 2). Of study population, the pooled OR were 1.15 (95% CI 1.03–1.29, *p* = .01) in general population, lower than acute ischemic stroke patients (OR = 1.60, 95% CI 1.14–2.25, *p* = .007) and hemodialysis patients (OR = 4.89, 95% CI 2.54–9.90, *p* < .001). Studies from Europe (OR = 1.05, 95% CI 0.91–1.20, *p* = .51) yielded lower OR of endpoints compared with Asia (OR = 1.89, 95% CI 1.38–2.60, *p* < .001) and United States (OR = 1.43, 95% CI 1.19–1.72, *p* < .001). Differences in PTFV1 measurement methods and study types seemed not affect study outcomes.

**Table 2:** Subgroup analysis

Subgroup	OR (95% CI)	*p* value	*I* ^2^ (%)
Area			
Asia(*n* = 2)	1.89 (1.38–2.60)	<.001	89.50
Europe(*n* = 3)	1.05 (0.91–1.20)	.506	24.51
United States(*n* = 4)	1.43 (1.19–1.72)	<.001	65.88
Measurement methods			
Manual(*n* = 3)	1.83 (1.01–3.36)	.050	88.33
Machine(*n* = 6)	1.18 (1.03–1.36)	.017	72.87
Study type			
Prospective(*n* = 7)	1.20 (1.07–1.33)	.001	80.51
Nonprospective(*n* = 2)	1.60 (1.14–2.25)	.007	64.50
Population			
General population(*n* = 6)	1.15 (1.03–1.29)	.010	64.02
Acute ischemic stroke patients(*n* = 2)	1.60 (1.14–2.25)	.007	64.50
Hemodialysis patients(*n* = 1)	4.89 (2.54–9.90)	‐	0

Abbreviations: CI, confidence interval; OR, odds ratios.

**Figure 4:**
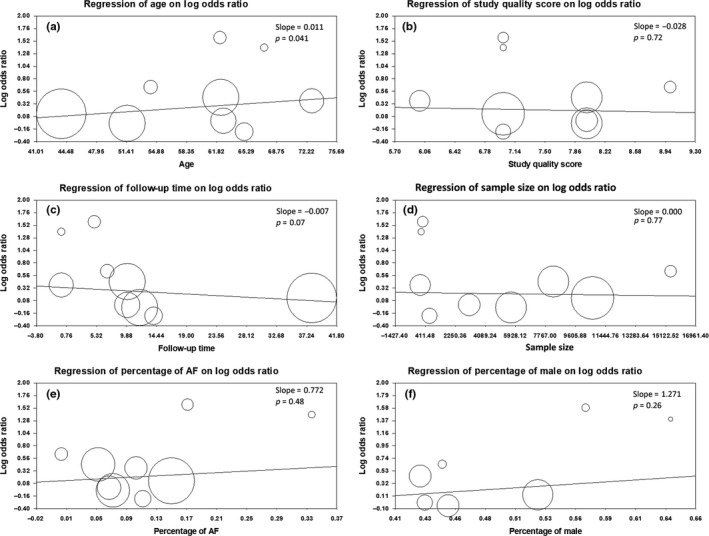
Meta‐regression plots of ORs of endpoints against continuous variables, including (a) age, (b) study quality score, (c) follow‐up time, (d) sample size, (e) percentage of AF, and (f) percentage of male

Meta‐regression analysis based on categorical variables showed that age (*p* = .041) was positively associated with OR of endpoint, while study quality score (*p* = .72), follow‐up time (*p* = .07), sample size (*p* = .77), percentage of AF (*p* = .48), and percentage of male (*p* = .26) were not predictive of study result (Figure 4). However, because of the relative small number of including studies, these results were only hypothesis‐generating rather than confirmatory.

## 4 DISCUSSION

Regarding to the controversy results from previous studies, this meta‐analysis demonstrated that PTFV1 as a common ECG marker was significantly associated with AF occurrence. As a categorical variable, abnormal PTFV1 increased 39% risk of AF occurrence. As a continuous variable, 1‐*SD* increased of PTFV1 increased 27% risk of AF occurrence. Sensitivity analysis in studies PTFV1 analyzed as a category variable showed that after excluding a study about hemodialysis patients, heterogeneity decreased and abnormal PTFV1 was still positively correlated to AF occurrence with a 23% increase in risk. Subgroup analysis revealed ORs in studies of general population or those in Europe were lower than the others.

Generally, detection of AF relied on electrocardiogram (ECG) or Holter. Persistent or permanent AF was convenient to diagnose but easily missed paroxysmal AF. However, hazard was equally no matter paroxysmal AF or permanent AF especially in causing stroke (Komatsu et al., 2010). Recently, long‐term noninvasive ambulatory ECG monitoring or insertable cardiac monitor (ICM) was used for screening AF in high‐risk patients (Gladstone et al., 2014; Sanna et al., 2014). Nevertheless, it was time‐consuming and burdensome for patients that its widespread use was restricted. Therefore, it was necessary to identify AF high‐risk population through some indicators. P wave indices derived from ECG including P wave duration, P wave dispersion, P wave area, P wave axis, and PTFV1 were quantitative measures of atrial electrical function (Magnani, Williamson, Ellinor, Monahan, & Benjamin, 2009). They were considered to be good predictors of AF (German, Kabir, Dewland, Henrikson, & Tereshchenko, 2016; Yoshizawa et al., 2014) but comprehensive medical evidences were relatively lack. Recently, a meta‐analysis conducted by Tse et al revealed that interatrial block (IAB) defined by a P wave duration > 120 ms was a significant predictor of both new‐onset AF and AF recurrence (Tse et al., 2018). Another meta‐analysis by Wang et al found prolonged P wave duration was strongly related to the risk of AF recurrence after radiofrequency catheter ablation (Wang, Chen, Li, Zhou, & Li, 2017). Comparing to other P wave indices, PTFV1 was easily visualized on ECG without requiring complex calculations, and thus, it can be a simple marker to identify AF high‐risk individuals. PTFV1 was considered to be a good predictor of stroke regardless of AF in a meta‐analysis by He et al (He et al., 2017). Our study was the first meta‐analysis explored the directly association of PTFV1 and AF risk. Eventually, we found PTFV1 was positively correlated with AF occurrence as both a categorical variable and continuous variable, indicating it might be a predictor of AF.

When PTFV1 firstly mentioned by Morris et al (Morris et al., 1964) in 1964, it was a concept representing left atrial (LA) overload in patients with various valvular heart diseases. Afterward, it was found to be meaningful not only in cardiovascular patients but also in general population. PTFV1 was composed of several factors including LA enlargement, LA hypertrophy increased, LA pressure, and abnormal interatrial conduction. AF development was associated with these structural alterations and electric remodeling (Goda et al., 2017; Ishida et al., 2010). Therefore, combining with our findings, we considered PTFV1 was a good marker of AF development.

Heterogeneities do exist among individual studies in our meta‐analysis due to the potentially clinical and methodological diversity. Sensitivity analysis found that a study conducted in hemodialysis patients contributed most to the heterogeneities. OR of PTFV1 and new‐onset AF in hemodialysis patients was much higher than other population (Nishi et al., 2013), indicating that PTFV1 was more useful in predicting AF occurrence in chronic volume overload patients. Subgroup analysis found different study area might be confounding factors for study outcomes. Studies conducted in Europe showed weaker association between PTFV1 and AF than studies conducted in Asia or United States. However, due to the small study number, this result might be occasional. Studies could be divided into three subgroups according to the study population including general population, acute ischemic stroke patients, and hemodialysis patients in this meta‐analysis. As mention above, hemodialysis patients had a much higher OR value of PTFV1 and AF. Meanwhile, OR in stroke patients was higher than general population. Since the relationship between PTFV1 and risk of incident ischemic stroke had already been demonstrated (He et al., 2017), it can be inferred that PTFV1 was also closely associated with AF occurrence in ischemic stroke patients. In general population, association between PTFV1 and AF was weaker but it was still significant. Age was found to be a confounding factor of the results according to meta‐regression analysis. It was understandable because advanced age had been proven to be the most prominent risk factor of AF occurrence in previous studies (Schnabel et al., 2015; Staerk, Sherer, Ko, Benjamin, & Helm, 2017). Other covariates including measurement methods, study types, study quality, follow‐up time, sample size, percentage of AF, and percentage of male did not affect the results. However, since the limiting number of included studies, these conclusions from subgroup and meta‐regression analysis were hypothesis‐generating rather than confirmatory.

Some limitations existed in this meta‐analysis. Number of included studies was relatively small, especially the studies that PTFV1 analyzed as a continuous variable. Although most of the studies were large‐scale prospective studies, there were still a small number of cross‐sectional or retrospective studies. Of the included studies, one of them was univariate analyses which gave the result as unadjusted OR, while others were multivariate analysis which attempted to control for confounders and gave adjusted ORs. Moreover, adjustment was not consistent across these studies, which made it difficult to assess the effect of confounding in a consistent manner. Finally, heterogeneities existed even sensitivity, subgroup, and meta‐regression analysis had been conducted. The sources of heterogeneities were not able to be fully explored or avoid, making it harder to make a definitive conclusion.

## 5 CONCLUSION

The present meta‐analysis revealed that PTFV1 derived from ECG, no matter as a category variable or a continuous variable, was significantly associated with the risk of AF. It was considered to be a good predictor of AF occurrence in population with or without cardiovascular diseases.

## CONFLICT OF INTEREST

The authors declare no potential conflict of interests.

## AUTHORS’ CONTRIBUTION

Jinlai Liu and Jieming Zhu raised the idea, designed the study and revised the article. Zhuoshan Huang and Zhenda Zheng extracted the information and performed quality assessment for each including study of the meta‐analysis, and write the article. Bingyuan Wu, Leile Tang, Xujing Xie, Ruimin Dong and Yanting Luo searched articles published and screened for the eligible study. Suhua Li performed the statistical analysis.

## ETHICAL APPROVAL

This article does not contain any studies with human participants or animals performed by any of the authors, and thus, no ethical approval is required.
